# Supplementary figures and images for: A signature of neural coding at human perceptual limits
Source: J Vis. 2016 Sep 7;16(11):4. doi: 10.1167/16.11.4 (PMC5024667; doi:10.1167/16.11.4)

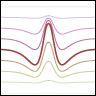

Supplement: Supplementary file 1 [file i1534-7362-16-11-4-icon01.gif]
